# Advancements in Buoy Wave Data Processing through the Application of the Sage–Husa Adaptive Kalman Filtering Algorithm

**DOI:** 10.3390/s23167298

**Published:** 2023-08-21

**Authors:** Sha Jiang, Yonghua Chen, Qingkui Liu

**Affiliations:** 1 Institute of Oceanology, Chinese Academy of Sciences, Nanhai Road No. 7, Shinan District, Qingdao 266071, China; mooring@qdio.ac.cn (S.J.); liuqk5102@qdio.ac.cn (Q.L.); 2 University of Chinese Academy of Sciences, Jingjia Road, Yanqi Lake Campus, Huairou District, Beijing 100049, China

**Keywords:** Sage–Husa adaptive kalman filter, combined filter, wave direction spectrum, acceleration sensor

## Abstract

In this paper, we propose a combined filtering method rooted in the application of the Sage–Husa Adaptive Kalman filtering, designed specifically to process wave sensor data. This methodology aims to boost the measurement precision and real-time performance of wave parameters. (1) This study delineates the basic principles of the Kalman filter. (2) We discuss in detail the methodology for analyzing wave parameters from the collected wave acceleration data, and deeply study the key issues that may arise during this process. (3) To evaluate the efficacy of the Kalman filter, we have designed a simulation comparison encompassing various filtering algorithms. The results show that the Sage–Husa Adaptive Kalman Composite filter demonstrates superior performance in processing wave sensor data. (4) Additionally, in Chapter 5, we designed a turntable experiment capable of simulating the sinusoidal motion of waves and carried out a detailed errors analysis associated with the Kalman filter, to facilitate a deep understanding of potential problems that may be encountered in practical application, and their solutions. (5) Finally, the results reveal that the Sage–Husa Adaptive Kalman Composite filter improved the accuracy of effective wave height by 48.72% and the precision of effective wave period by 23.33% compared to traditional bandpass filter results.

## 1. Introduction

The precise measurement and real-time monitoring of marine environments are of paramount importance for scientific research, ocean engineering, and marine disaster early warning systems [1]. In this context, waves, as a significant parameter of marine environments, wield profound influence over areas such as ship design, marine engineering construction, and oceanic environment warnings [2]. Consequently, the accurate and rapid acquisition and processing of wave parameters represents a pressing task in our current landscape [3].

However, given the complex and mutable nature of marine environments, coupled with errors inherent in wave sensors and the influence of external environmental noise, the wave data we obtain often contain significant errors [4]. As a result, extracting true wave information amidst the noise presents a substantial challenge in contemporary marine engineering and oceanographic research. In traditional wave measurement data processing, the predominant methods for eliminating interference signals from raw data collected by accelerometers encompass the summation-and-averaging approach and the low-pass filtering technique.

In 2019, scholar Shoujun Wang [5] from Tianjin University in China employed the sliding average method to integrate a certain length of raw acceleration signals and fit the result into a sliding average trend line. By subtracting this trend line from the signal-to-be-processed, stable and smooth data were obtained. However, this method has proven less effective when dealing with abrupt signal changes, potentially leading to loss and distortion of original data features.

In 2022, researcher Zhuoya Deng [6] from the China National Marine Technology Center found that when dealing with large volumes of acceleration signal data, the summation-and-averaging method was not only cumbersome and labor-intensive, but was also prone to incidental errors during the computation process.

Similarly, low-pass filtering has its limitations. In 2023, scholar Giovanni Battista Rossi [7] from Italy utilized a bandpass filter to process the signals measured by the Alghero buoy. Although setting appropriate upper and lower cutoff frequencies effectively filtered out zero-offset errors and low-frequency random noise, the fixed cutoff frequency revealed its significant shortcoming when sea conditions changed and the distribution of noise frequencies altered, namely its inability to adapt to noise frequencies under varying sea conditions.

From this, it is clear that these traditional methods have limitations in processing abrupt signal changes and adapting to the variability of sea conditions. In contrast, the Sage–Husa Adaptive Kalman filtering algorithm, an established method of linear optimal estimation, effectively manages and mitigates these noise disturbances, offering optimal system state estimations [8]. Its prediction–update iterative process continually refines system state estimates, hence providing immense practical value in the field of marine environment monitoring, which requires real-time processing and state estimation.

This paper will primarily investigate the filtering process of wave acceleration data collected by nine-axis attitude sensors, utilizing a combined filtering algorithm based on the Sage–Husa Adaptive Kalman filter. By subsequently integrating and solving the filtered data, we aim to attain more accurate wave parameters such as wave height, wave period, and wave direction spectrum [9]. This approach promises to improve the processing accuracy of wave sensor data, providing more accurate data support for real-time marine environment monitoring and prediction.

Through this research, we aspire to further propel the application of the Kalman filter within the field of marine science, enhance the accuracy of wave parameter measurement and processing, and provide more precise, real-time data support for marine scientific research and ocean engineering. Simultaneously, by studying the filtering algorithm, we aim to deepen our understanding of the errors associated with wave sensors and their handling methods, thus providing a theoretical foundation for the further development of related technologies.

## 2. Materials and Methods

### 2.1. Kalman Filtering

The Kalman Filter is a Linear Quadratic Estimation (LQE) method that is widely used in various fields, including navigation [10], signal processing [11], control systems [12], biological tissue identification [13], etc. The Kalman Filter is based on the state–space model, and for linear systems and Gaussian noise, this model is usually represented by the following two equations [8]:

- The state equation: this describes the dynamic evolution of the system state, usually a linear or linearized differential equation.
  (1) $$x_{k}=Ax_{k-1}+Bu_{k-1}+w_{k-1}$$
- The observation equation: this describes how to obtain observation data from the system state.
  (2) $$z_{k}=Hx_{k}+v_{k}$$

Here, $x_{k}$ is the state at *k* time, $u_{k}$ is the control input at *k* time, $z_{k}$ is the observation at *k* time, $w_{k-1}$ is the process noise, $v_{k}$ is the observation noise, and they are all assumed to be Gaussian distributed. *A* is the system state transition matrix, *B* is the control input matrix, and *H* is the observation matrix.

In order to successfully apply the classic Kalman filter, the following conditions must be met [14]:

1. The mean and covariance matrix of the initial state estimation are known.
2. The process noise and measurement noise are composed only of white noise, meaning they respectively satisfy Gaussian density functions with variances *Q* and *R*.
  (3) P(w)~N(0,Q)
  (4) P(v)~N(0,R)

The Kalman Filter can be implemented through a recursive algorithm where the state is considered as a Gaussian variable and is described through the following two steps: prediction and update.

A. Prediction Step [11]

In the Kalman Filter, the prediction step is first performed, using the state equation to predict the next state and covariance. The prediction value ${\dot{x}}_{k}^{\prime }$ and the error covariance matrix $P_{k}^{\prime }$ between the predicted value and the true value are first calculated.

(5) $${\dot{x}}_{k}^{\prime }=A{\dot{x}}_{k-1}+Bu_{k-1}$$

(6) $$P_{k}^{\prime }=AP_{k-1}A^{T}+Q$$

B. Update Step

After prediction, the filter uses newly collected observation data to correct the predicted state, which is the update step. In this step, the Kalman Gain *K* is calculated, which determines the trade-off between the prediction and the observation.

(7) $$K_{k}=P_{k}^{\prime }{H^{T}(HP_{k}^{\prime }H^{T}+R)}^{-1}$$

Then, the state of the estimated value ${\dot{x}}_{k}$ is updated using the Kalman Gain and the observation residual (the difference between the observation value and the predicted value).

(8) $${\dot{x}}_{k}={\dot{x}}_{k}^{\prime }+K_{k}(z_{k}-H{\dot{x}}_{k}^{\prime })$$

Finally, the error covariance matrix $P_{k}$ between the estimated value and the true value is calculated to prepare for the next recursion.

(9) $$P_{k}=(I-K_{k}H)P_{k}^{\prime }$$

The key to the Kalman Filter is that it iterates through this predict–update process, improving the system state estimation with each new observation received. This allows the Kalman Filter to effectively handle noise and uncertainty, providing an optimal estimation of the system state.

### 2.2. Sage–Husa Adaptive Kalman Filtering

An adaptive Kalman filter estimates and corrects the model and noise statistical characteristics while using the measurement data, thereby modifying the filter design and filter error.

Known from Formulas (1) and (2), $w_{k}$ represents process noise and $v_{k}$ represents observational noise, defined as:

(10) $$E[w_{k}]=q_{k}$$

(11) $$E[w_{k}w_{k}^{T}]=Q_{k}$$

(12) $$E[v_{k}]=r_{k}$$

(13) $$E[v_{k}v_{k}^{T}]=R_{k}$$

Adaptive control requires updates to $q_{k}$, $Q_{k},\;r_{k}$, and $R_{k}$ [15]:

(14) $${\dot{x}}_{k}^{\prime }=A{\dot{x}}_{k-1}^{\prime }+Bu_{k-1}+H{\dot{q}}_{k-1}$$

(15) $$P_{k}^{\prime }=AP_{k-1}A^{T}+B{\dot{Q}}_{k-1}B^{T}$$

where ${\dot{q}}_{k-1}$ and ${\dot{Q}}_{k-1}$ are the initial estimated values.

Like the Kalman filter, the purpose of the gain value of the adaptive Kalman filter is to adjust the ratio between ${\dot{x}}_{k}^{\prime }$ and $z_{k}-{\dot{x}}_{k}^{\prime }$ [16].

Through derivation, we obtain:

(16) $$K_{k}=P_{k}^{\prime }H^{T}{\left(HP_{k}^{\prime }H^{T}+{\dot{R}}_{k-1}\right)}^{-1}$$

Residual:

(17) $$\epsilon_{k}=z_{k}-H{\dot{x}}_{k}^{\prime }-{\dot{r}}_{k-1}$$

Therefore, the estimated value is:

(18) $${\dot{x}}_{k}={\dot{x}}_{k}^{\prime }+K_{k}(z_{k}-H{\dot{x}}_{k}^{\prime }-{\dot{r}}_{k-1})$$

(19) $$P_{k}=(I-K_{k}H)P_{k}^{\prime }$$

The update formulas for $q_{k}$, $Q_{k},\;r_{k}$ and $R_{k}$ are as follows [17]:

(20) $${\dot{q}}_{k}=[1-d(t-1)]{\dot{q}}_{k-1}+d(t-1)({\dot{x}}_{k}-H{\dot{x}}_{k-1})$$

(21) $${\dot{Q}}_{k}=[1-d(t-1)]{\dot{Q}}_{k-1}+d(t-1)(K_{k}\epsilon_{k}\epsilon_{k}^{T}K_{k}^{T}+P_{k}-AP_{k-1}A^{T})$$

(22) $${\dot{r}}_{k}=[1-d(t-1)]{\dot{r}}_{k-1}+d(t-1)({\dot{z}}_{k}-H{\dot{x}}_{k}^{\prime })$$

(23) $${\dot{R}}_{k}=[1-d(t-1)]{\dot{R}}_{k-1}+d(t-1)(\epsilon_{k}\epsilon_{k}^{T}-HP_{k}^{\prime }H^{T})$$

where d(t)=1/t or $d(t)=(1-b)/(1-b^{t+1})$, (0 < *b* < 0), *b* is referred to as the forgetting factor, and the value of *d*(*t*) is between 0.95 and 0.99. If *d*(*t*) equals 0, the adaptive Kalman filter will transform into a Kalman filter.

### 2.3. Exploring Adaptive Filtering

Adaptive filtering algorithms have been extensively studied. For instance, there exist methods such as the Extended Kalman Filter (EKF) based on the online soft tissue characteristics of the Hunt–Crossley contact model [18], and the distributed optimal fusion method based on the Cube Law, which is used for the identification and prediction of kinematic model error for integrated UAV navigation [19]. The Extended Kalman Filter demonstrates superior performance in nonlinear systems, particularly in its adaptability to complex dynamic environments.

Applying the theory of stochastic processes, waves can be considered as the superposition of individual waves with different phases, frequencies, and amplitudes [20]. Thus, we have chosen to use the Sage–Husa adaptive Kalman filter in this experiment. The Sage–Husa adaptive Kalman filter proves effective in handling linear systems and exhibits unique advantages in our research environment.

Firstly, the Sage–Husa adaptive Kalman filter introduces an adaptive factor *b*, which can correct noise parameters in real-time. This real-time characteristic is crucial when dealing with wave data that is continuously generated and may vary in statistical properties. Secondly, while the Sage–Husa adaptive Kalman filter is still based on linear assumptions, its adaptive nature allows its performance to rival that of nonlinear filters in certain circumstances. Additionally, the real-time and converging characteristics of this filter demonstrate superior performance in handling ocean wave data.

However, the distributed optimal fusion method based on the Cube Law, while showing significant advantages in processing information from multiple sensors, is somewhat limited in our research as we only deal with vertical acceleration data of ocean waves collected by a single accelerometer.

In conclusion, we have selected the Sage–Husa adaptive Kalman filter as the primary research method in this experiment mainly due to its advantages in handling ocean wave data. However, we acknowledge that the exploration of other filtering methods may be necessary for the processing of nonlinear systems. In future research, we plan to explore the application of these methods in our research environment.

## 3. Wave Parameter Analysis

This study employs a wave buoy based on an accelerometer for analysis. In the process of the buoy following wave movement, the accelerometer outputs real-time acceleration signals reflecting the characteristics of wave motion. By applying a second integration to the discrete *Z*-axis vertical acceleration signals collected by the processor, the displacement of water quality points is accurately estimated [21]. The wave height and period are typically defined based on the zero-crossing method in the wave motion trajectory. By combining the acceleration data from the X and Y axes with the data from the triaxial magnetometer and gyroscope, the direction of wave motion is calculated. Finally, the wave direction spectrum is derived from the wave displacement signals and wave direction signals. This workflow constructs a comprehensive system for wave measurement and analysis, providing robust data support for marine wave dynamics research.

**Figure 1:**
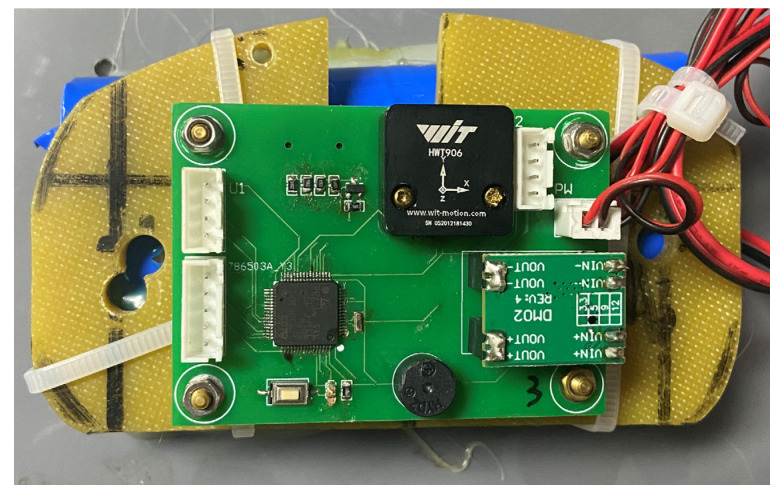
Lab-developed acceleration wave buoy based on micro-electro-mechanical systems (MEMS) attitude sensors.

In Figure 1 below, a sample diagram of a wave gauge, developed in-house by the laboratory, is presented.

### 3.1. Acceleration Conversion

First, it is necessary to use the quaternion method to convert the acquired acceleration signal because, during wave measurement, the sensor collects the original wave signal based on the carrier coordinate system $B:OX_{b}Y_{b}Z_{b}$, while the acceleration signal for wave inversion is based on the navigation coordinate system $N:OX_{n}Y_{n}Z_{n}$.

The rotation of the carrier coordinate system relative to the navigation coordinate system can be represented by rotation quaternions *Q*, that is:

Let’s define a quaternion as follows:

(24) $$Q(q_{0},q_{1},q_{2},q_{3})=q_{0}+q_{1}i+q_{2}j+q_{3}k$$

Its conjugate complex number can be represented as:

(25) $$Q^{*}(q_{0},q_{1},q_{2},q_{3})=q_{0}-q_{1}i-q_{2}j-q_{3}k$$

The coordinate change matrix from the carrier coordinate system *B* derived from the quaternion to the navigation coordinate system *N* is as follows [22]:

(26) $$C_{b}^{R}=\left[\begin{array}{ccc} q_{0}^{2}+q_{1}^{2}-q_{2}^{2}-q_{3}^{2} & 2(q_{1}q_{2}-q_{0}q_{3}) & 2(q_{1}q_{3}+q_{0}q_{2})\\ 2(q_{1}q_{2}+q_{0}q_{3}) & q_{0}^{2}-q_{1}^{2}+q_{2}^{2}-q_{3}^{2} & 2(q_{2}q_{3}-q_{0}q_{1})\\ 2(q_{1}q_{3}-q_{0}q_{2}) & 2(q_{2}q_{3}+q_{0}q_{1}) & q_{0}^{2}-q_{1}^{2}-q_{2}^{2}+q_{3}^{2} \end{array}\right]$$

### 3.2. Principles of Wave Height and Wave Period Measurement

The wave displacement data can be obtained by performing a second integration on the acceleration data after coordinate transformation. Integration in the frequency domain involves using the interchangeability of the sine and cosine integrals of the acceleration signal. This method can effectively prevent the small DC component errors in the time domain signal from being amplified during integration [23].

The numerical calculation formula for the second integral in the frequency domain is [24]:

(27) $$y(r)=\sum_{k=0}^{N-1}\;-\;\frac{1}{{\left(2\pi k\Delta f\right)}^{2}}H(k)X(k)e^{j2\pi kr/N}$$

where:

(28) $$H\left(k\right)=\left\{\begin{array}{c} 1,(f_{d}\;\leq \;k∆f\;\leq \;f_{u})\\ 0,\;(else) \end{array}\right.$$

The discrete Fourier transform of the input signal *x(r)* is *X(k)*, the frequency response function of the filter is *H(k)*, the lower cutoff frequency of the filter is $f_{d}$ the upper cutoff frequency is $f_{u}$, and the frequency resolution is ∆f. After integrating in the frequency domain, the water quality point motion trajectory in the time domain can be obtained through the fast Fourier inverse transform.

### 3.3. Principle of Wave Direction Measurement

This paper uses a combination of a three-axis accelerometer and magnetometer to measure wave direction, calculating the composite acceleration direction of the buoy’s horizontal *X*-axis and *Y*-axis at each zero-crossing point as the relative direction of wave motion. The X and Y horizontal plane is divided evenly into 16 sectors with a 22.5° interval. Using statistics, the frequency of wave direction distribution in each sector is calculated. The direction with the highest distribution frequency is the main wave direction. Count the number of waves in each sector as n, and the total number of waves as *N*, so the frequency of wave direction in each interval is:

(29) $$\eta =\frac{n}{N}\;\times \;100\%$$

Establish a coordinate system with the *X*-axis and *Y*-axis of the inertial sensor. The angle α between the direction of the composite horizontal vector acceleration and the *Y*-axis is the relative wave direction. Combining with the true magnetic heading obtained from the magnetometer, the true geographic wave direction can be obtained. The calculation of the angle α has different algorithms depending on the quadrant. Let us assume $A_{X}$ is the *X*-axis acceleration and $A_{Y}$ is the *Y*-axis acceleration [25].

(30) $$A_{Y}=0,\;A_{X}>0,\;\alpha =90,\;A_{X}<0,\;\alpha =270$$

(31) $$A_{X}=0,\;A_{Y}>0,\;\alpha =0,\;A_{Y}<0,\;\alpha =180$$

(32) $$A_{X}>0,\;A_{Y}>0,\;\alpha =arctan(\frac{A_{X}}{A_{Y}})$$

(33) $$A_{X}>0,\;A_{Y}<0,\;\alpha =180-arctan(\frac{{-A}_{X}}{A_{Y}})$$

(34) $$A_{X}<0,\;A_{Y}>0,\;\alpha =360-arctan(\frac{{-A}_{X}}{A_{Y}})$$

(35) $$A_{X}<0,\;A_{Y}<0,\;\alpha =180+arctan(\frac{A_{X}}{A_{Y}})$$

**Figure 2:**
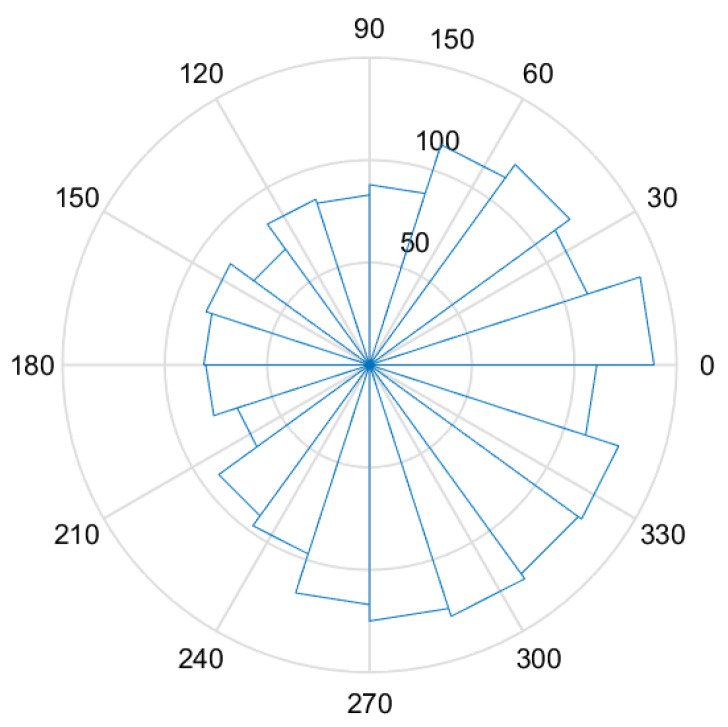
Direction rose diagram. The radius of each blue sector represents the number of occurrences within that angular range.

As shown in Figure 2, it presents a directional distribution diagram of waves, also known as the directional rose diagram, calculated from one set of original acceleration and angle data obtained from a turntable experiment of simulated sine waves (as detailed in Section 5).

### 3.4. Wave Energy Spectrum

The wave spectrum data stored and processed in the computer are all discrete. The discrete wave spectrum represents the distribution of wave energy per unit frequency interval as a function of frequency.

This paper employs the improved windowed average periodogram method (Welch’s method [7]) to calculate the wave energy spectrum. The periodogram method calculates the wave spectrum data by performing a Fast Fourier Transform (FFT) on the wave surface displacement, considering the symmetry of the double-side spectrum, and then smoothing it.

The relationship between the simulated wave displacement *x(t)* and the wave spectrum is as follows [6]:

(36) $$\tilde{S}(f)=\frac{1}{N}{\left|[FFT(x(t))]\right|}^{2}$$

(37) $$\tilde{S}\left(k\right)=\frac{1}{N}{\left|X\left(k\right)\right|}^{2}=\frac{1}{N}{\left|\left[FFT\left(x\left(n\right)\right)\right]\right|}^{2},\;k=0,1,2,\ldots$$

In the 1950s, overseas scholars first applied the theory of radio noise to wave research. Since then, spectra have been used to describe the random processes of fluctuating sea surfaces, which has become a primary research method. To date, many models describing waves have been proposed, viewing the sea waves as a stationary normal process with ergodicity. Among them, the Phillips spectrum, PM (Pierson and Moskowitz, abbreviated as PM) spectrum, JONSWAP (Joint North Sea Wave Project, abbreviated as JONSWAP) spectrum, etc., are relatively famous and have been widely used in the fields of marine science and marine engineering [6].

**Figure 3:**
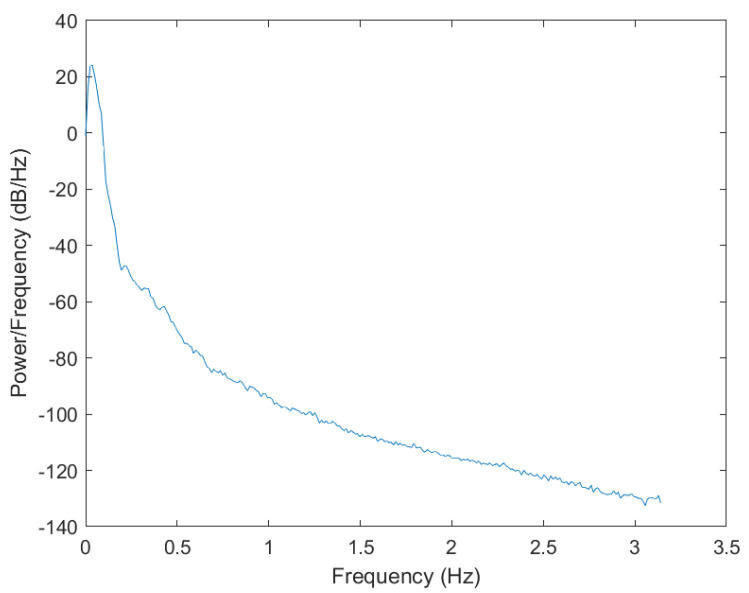
Wave power spectral density diagram.

As depicted in Figure 3, this is a graph obtained from calculating the wave power spectral density from the displacement data inverted from the PM spectrum.

The basic principle of the Welch’s method is to use a finite length sample sequence for a Fast Fourier Transform to estimate or approximately represent the power spectrum of a random sequence. Errors inevitably occur during the calculation process, and factors such as the number of sampling points, sampling frequency, and the overlap rate of segmented signals can all affect the precision of the results.

### 3.5. Wave Direction Spectrum

Applying the theory of random processes, waves can be seen as composed of superpositions of single waves with different phases, frequencies, and amplitudes, which combine to form a wavefront *x(t)* describing the displacement of sea waves at a fixed point [18].

(38) $$x(t)=\sum_{n=1}^{\infty }a_{n}cos(\omega_{n}t+\varepsilon_{n})$$

In the above formula, $a_{n}$ represents the amplitude of the constituent waves, $\omega_{n}$ represents the circular frequency, and $\varepsilon_{n}$ represents the initial phase randomly distributed in 0–2π.

The wave spectrum plays a significant role in studying the evolution of wave motion and is also an important physical quantity describing the internal structure of waves. A one-dimensional spectrum, or wave energy spectrum, displays the distribution of wave energy size at different frequency components. A two-dimensional spectrum, or wave direction spectrum, can describe the distribution of wave energy size and direction relative to frequency simultaneously.

The estimation of the wave energy spectrum is derived from the characteristics of wave displacement and can only represent the distribution of wave energy with respect to frequency, lacking a description of the distribution of wave direction and frequency. The estimation of the wave direction spectrum requires the use of the Fourier series method, and its theoretical basis is the cross-spectrum of the power between two wave characteristics, which equals the Fourier transformation of the product of the transfer function and the wave direction spectrum between the wave surfaces [6].

(39) $$\varphi_{mn}(f)=\int_{-\pi }^{\pi }H_{m}(f,\theta )\bar{H_{n}}(f,\theta )exp(-ikx_{mn})S(f,\theta )d\theta$$

In the formula, $\varphi_{mn}(f)$ is the cross-spectrum of two wave characteristics *m*, *n*; is the cross-spectrum of two wave characteristics *m*, *n*; $H_{m}(f,\theta )$ is the transfer function of wave characteristics *m* between wave surfaces; ${\bar{H}}_{n}(f,\theta )$ is the conjugate function of $H_{n}(f,\theta )$; *k* is the wave number vector; $x_{mn}$ is the vector transformed from wave characteristics *m* to *n*; and S(f,θ) is the wave direction spectrum represented by frequency *f* and propagation direction θ.

Suppose the wave direction spectrum S(f,θ) can be expanded into a Fourier series [26]:

(40) $$S(f,\theta )=\frac{a_{0}(f)}{2}+\sum_{n=0}^{2}\left(a_{n}(f)cos(n\theta )+b_{n}(f)sin(n\theta )\right)$$

The acceleration signal measured by the sensor can integrate the displacement characteristics in three directions, so the Fourier series is only expanded to the second term, $a_{0},\;a_{1},\;b_{1},\;{a_{2},\;b}_{2}$ sed to represent the coefficients of the direction spectrum. From this, the first five coefficients of the Fourier series can be solved [27]:

(41) $$\begin{array}{c} a_{0}=\frac{C_{11}}{\pi },\;a_{1}=\frac{Q_{12}}{\sqrt{(C_{22}+C_{33})C_{11}}},\;b_{1}=\frac{-Q_{13}}{\sqrt{(C_{22}+C_{33})C_{11}}},\\ a_{2}=\frac{C_{33}-C_{22}}{C_{33}+C_{22}},\;b_{2}=\frac{-C_{11}}{C_{33}+C_{22}} \end{array}$$

$C_{mn}$ represents the co-phase spectrum, and $Q_{mn}$ represents the orthogonal spectrum.

The spectral width of the wave directional spectrum estimated by the Fourier series method is generally quite wide, and when it is asymmetrically distributed, its description of sea wave characteristics is relatively poor. Furthermore, due to the influence of marine noise, certain errors will also occur in its calculation results.

## 4. Simulate

**Figure 4:**
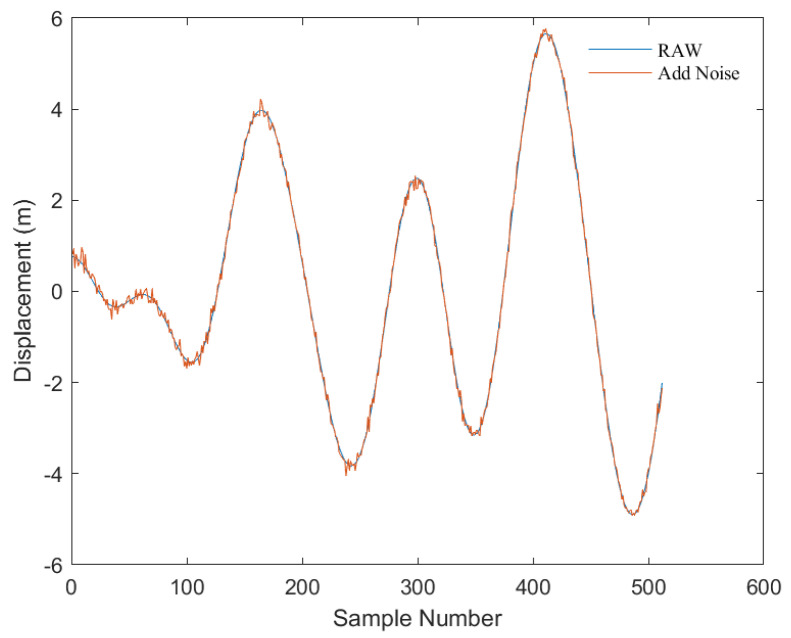
Comparison of original displacement signal and noise-added displacement signal. The blue line represents the original displacement signal calculated from the PM spectrum, and the red line represents the displacement signal after adding noise. The *x*-axis represents the number of sampling points, which total 512 displacement data points with a time interval of 0.1 s between each point; the *y*-axis represents the displacement value (unit: m).

As depicted in Figure 4, this is a comparison before and after the addition of Gaussian-distributed random noise (with a mean of 0, variance of 0.1, and array size identical to the original displacement signal) to the displacement signals calculated from the PM spectrum.

**Figure 5:**
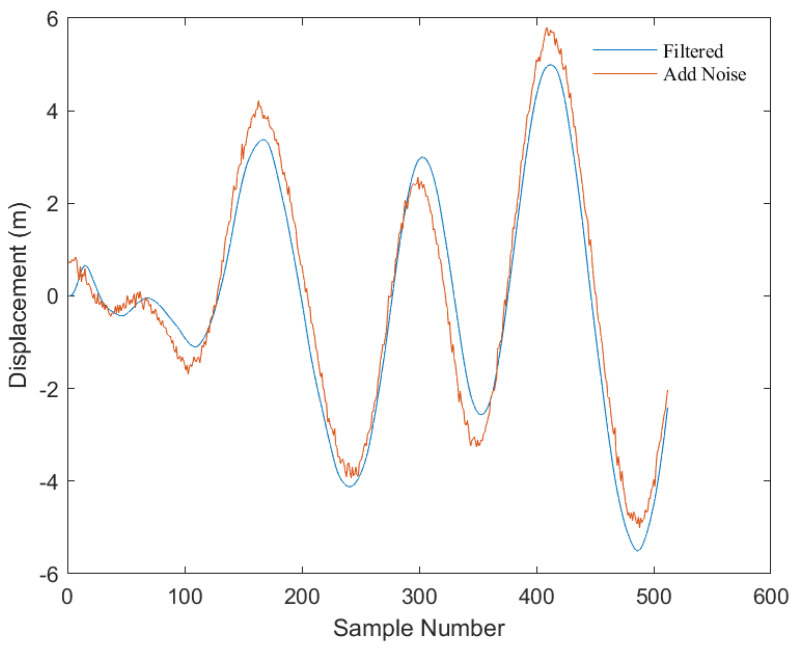
Comparison before and after bandpass filtering.

As shown in Figure 5, this is a comparison of displacement signals with added random noise before and after Bandpass filtering (in this experiment, the cutoff frequencies of the Bandpass filter were set to 0.01 Hz–0.5 Hz).

**Figure 6:**
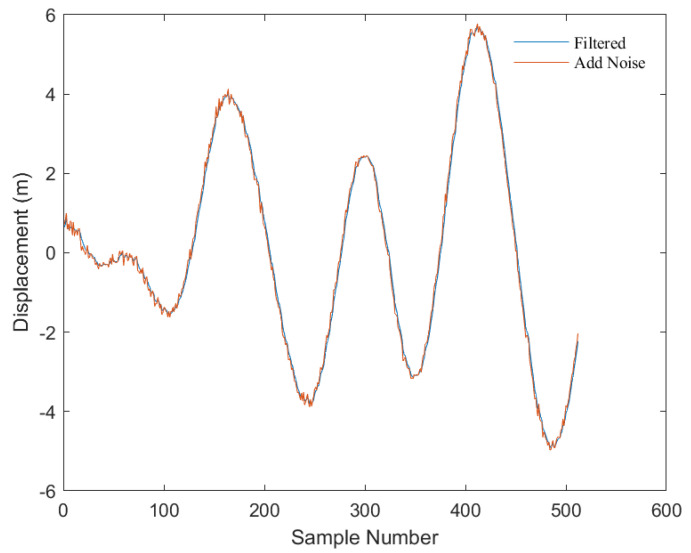
Comparison before and after Sage−Husa Adaptive Kalman filtering.

As shown in Figure 6, here is a comparison before and after applying the Sage–Husa adaptive Kalman filter to the noise-added signal.

**Figure 7:**
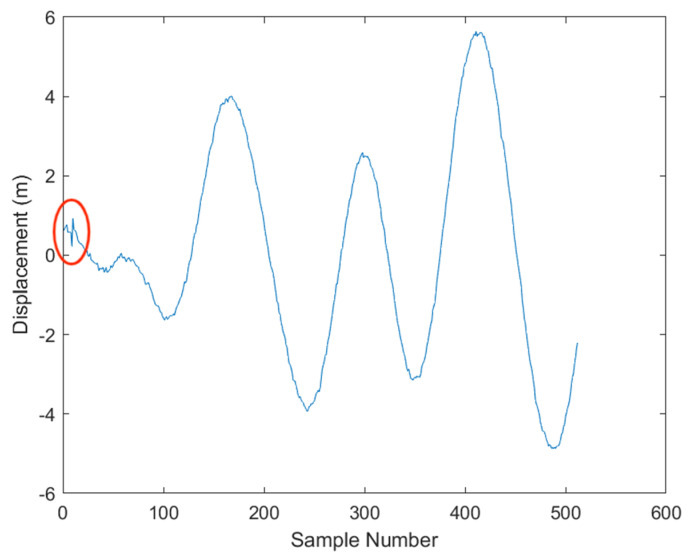
Graph after Sage−Husa Adaptive Kalman filtering. The red circle denotes the adaptive learning process of the Kalman filter.

As shown in Figure 7, to observe the effects of the Sage–Husa adaptive Kalman filter more directly, the following graph is presented after the filtering.

Based on the above, graphical analysis shows that the pure bandpass filter has stringent requirements for the selection of upper and lower cut-off frequencies. However, the actual sea conditions are unpredictable, and it is not easy to modify the upper and lower cut-off frequencies of the bandpass filter in real-time. The Sage–Husa adaptive Kalman filter compensates for this shortcoming perfectly. It only requires an initial state and can achieve satisfactory filtering results through continuous adaptive feedback and minimum error computation.

**Figure 8:**
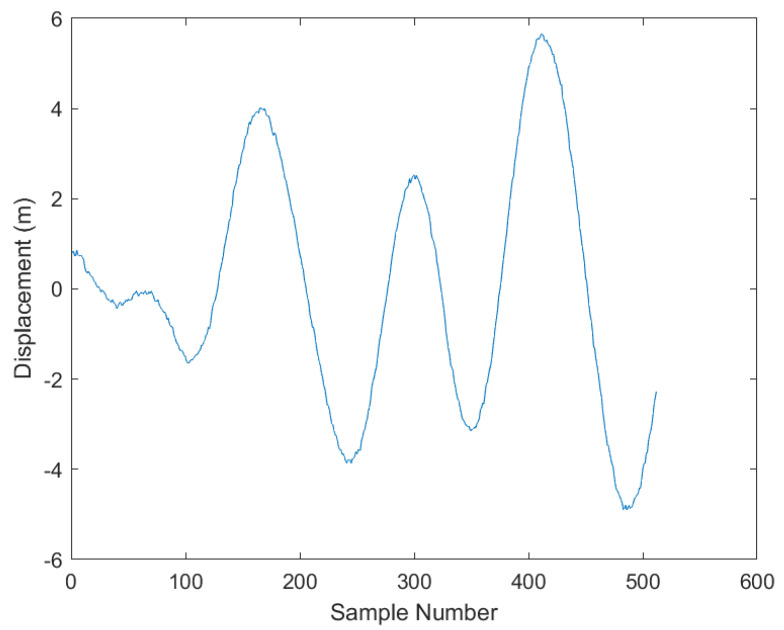
Training process of the Sage−Husa Adaptive Kalman filter.

However, the Sage–Husa Adaptive Kalman filter has some shortcomings as well [17]. During the initial stage, it needs a certain amount of time to self-train the filter parameters, which may lead to some deviations. The solution to this is to pre-start the filter, allowing it to operate for a while before formal filtering begins, as shown in Figure 8.

In Table 1 below, it lists the effective wave heights and effective wave periods of the wave displacement signals calculated through the PM spectrum. The table also includes the specific values of effective wave heights and effective wave periods obtained after processing through Bandpass filtering and Sage–Husa adaptive Kalman combined filtering.

From the results, it is evident that in this simulation experiment, the performance of the Sage–Husa adaptive Kalman filter is superior to that of the Bandpass filter.

In the actual measurement process, due to the presence of zero offset error in the accelerometer and the influence of low-frequency random noise on the sea’s acceleration data [28], the impact will be amplified countless times when performing a second integration on the original acceleration signal. These errors and noises are mainly distributed around 0 Hz in the frequency domain, and since the Sage–Husa Adaptive Kalman filter uses a method of filtering time-domain signals, its processing effects on low-frequency signals and zero-bias error are not optimal.

Therefore, this study designs a composite filtering method that combines the Sage–Husa Adaptive Kalman filter and the Butterworth band-pass filter, specifically for handling low-frequency signals.

**Figure 9:**
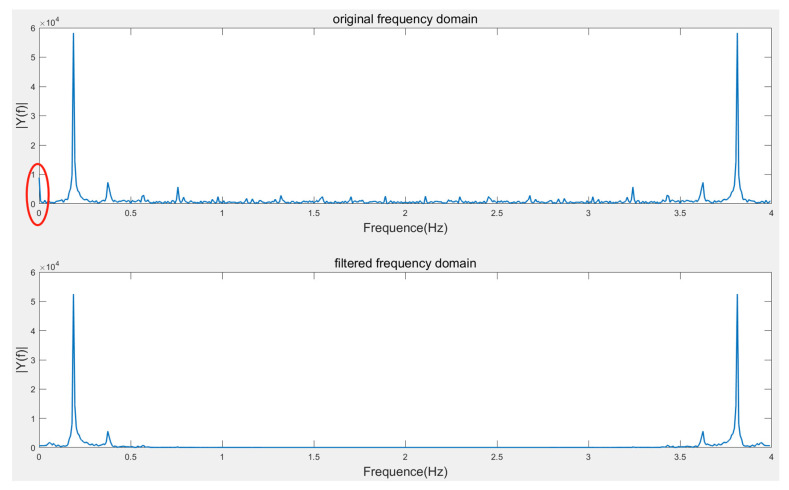
Comparison of spectra before and after combined filtering. The red circle denotes the zero−bias error.

**Figure 10:**
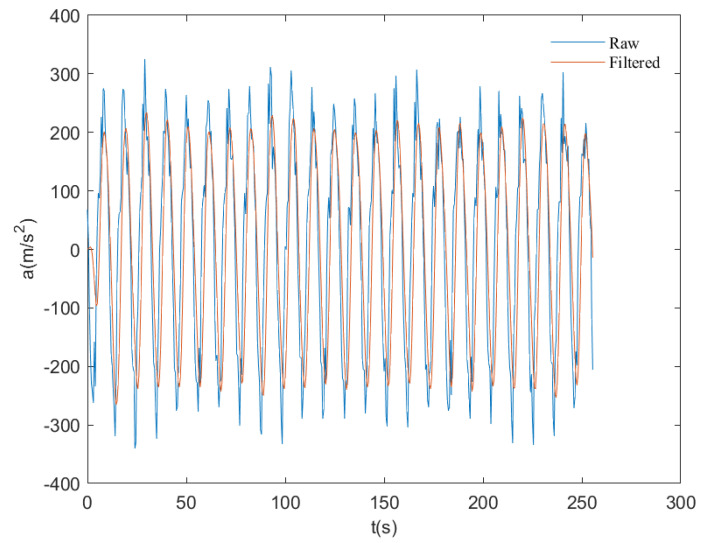
Comparison of waveforms before and after combined filtering.

As depicted in Figure 9, it is a spectrum comparison before and after filtering a set of simulated wave acceleration data obtained from the turntable experiment:

As shown in Figure 10, it demonstrates a waveform comparison before and after filtering for the same set of acceleration data:

## 5. Experiments and Results

Applying the theory of stochastic processes, waves can be considered as the superposition of multiple individual waves with distinct phases, frequencies, and amplitudes [20]. To validate the accuracy of the wave analysis algorithm under different wave heights and periods, we built a simple wave simulator in our laboratory, used to mimic the up and down motion of a wave gauge on the sea surface. This device operates by controlling two sets of mechanical arms to rotate at a uniform speed. The motion along the *z*-axis can be approximated as a standard sinusoidal motion. Furthermore, twice the length of the mechanical arm equates to the wave height of the sinusoidal motion, while the time it takes for the mechanical arm to make a full rotation corresponds to the period of the sinusoidal motion.

**Figure 11:**
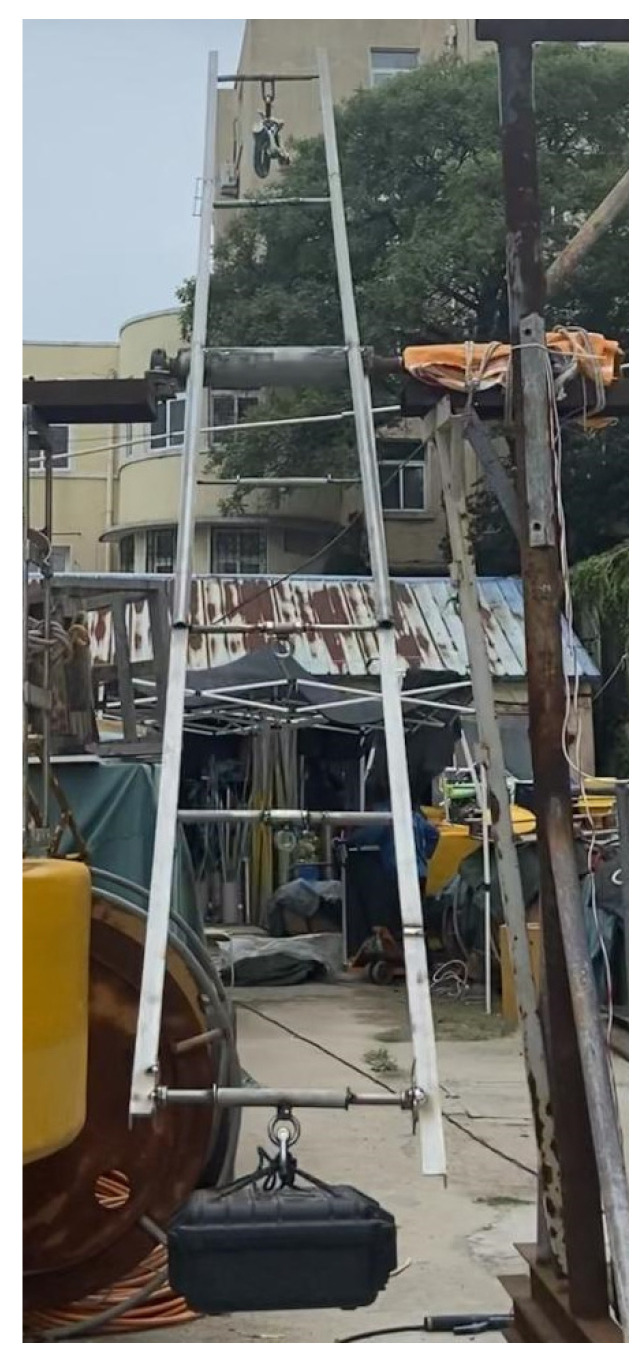
It depicts the actual scene of the turntable experiment simulating sinusoidal wave motion. There are support frames on both sides of the picture, with a rotating shaft mounted in the middle. The rotating shaft operates via the drive mechanism, which is connected to a computer on the shelf to the right of the picture through a data cable to control the rotation speed. The rotating shaft drives the upper and lower sets of mechanical arms to simulate the sinusoidal motion of waves. The mechanical arm at the bottom of the picture has four crossbeams, capable of simulating wave motion with four different motion radii of 2.00 m, 1.50 m, 1.05 m, and 0.55 m. A black insulation box is hung on one of the crossbeams, housing a MEMS nine-axis accelerometer. Simultaneously, corresponding unload buckles are hung on the mechanical arm at the top of the picture to balance the mechanical arms, ensuring uniform rotation of the two sets of mechanical arms.

As depicted in Figure 11, this is an actual image of the wave simulation apparatus.

An electric motor is used as a driving device to rotate the mechanical arm, which is equipped with a rotating shaft. The wave gauge can make approximate sinusoidal motion in the vertical direction with the rotating shaft. The wave simulation device has two sets of replaceable mechanical arms, each equipped with two rotating shafts of different radii. This device can simulate wave motion with four different wave heights of 4.00 m, 3.00 m, 2.10 m, and 1.10 m. The motor speed is regulated by controlling the motor voltage through a stabilizer, supporting a simulated wave period of 5–11 s.

Four different wave heights were chosen, each corresponding to three different wave periods for the experiments. The standard wave height, also referred to as the significant wave height in this turntable experiment simulating sinusoidal wave motion, is determined by the length of the mechanical arm and is equal to twice its length. This value serves as a standard reference for the experiment, compared with the effective wave height calculated by the filtering algorithm, thereby assessing the precision of the filtering algorithm. The time taken for the mechanical arm to rotate 10 circles is recorded using a stopwatch, and the average value is taken as the standard period. Before the experiment, the wave gauge was calibrated and the parameters were set, with a sampling frequency of 4 Hz and a sampling length of 2048.

There was a total of 12 sets of experiments, each repeated three times. The experimental simulation wave parameters are shown in the following Table 2:

According to the “People’s Republic of China National Standard—Seashore Observation Specification”, the error analysis formula is:

(42) $$H_{error}=\frac{\left|H_{1/3}-H_{0}\right|}{H_{0}}\;\times \;100\%$$

(43) $$T_{error}=|T_{1/3}-T_{0}|$$

where $H_{1/3}$ is the effective wave height, $H_{0}$ is the standard wave height, $T_{1/3}$ is the effective wave period, and $T_{0}$ is the standard wave period. Based on Formulas (42) and (43), error analysis was conducted on 36 sets of experiments.

**Figure 12:**
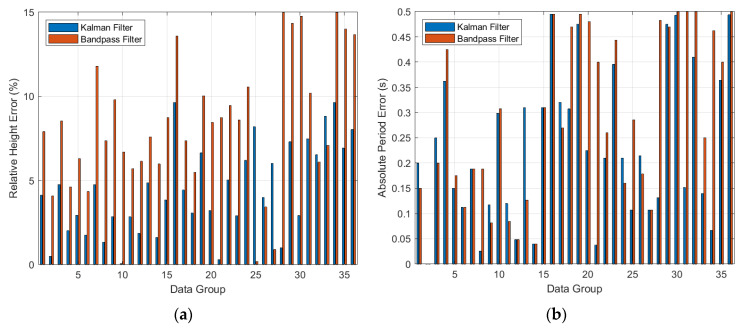
(**a**) It displays the comparison of the relative error in significant wave height obtained from 36 sets of simulated sine wave turntable experiments. The *x*-axis represents the experiment groups, while the *y*-axis represents the percentage of relative error in significant wave height (unit: %). In the figure, the error percentages calculated using the Sage–Husa adaptive Kalman combined filter are represented by blue bar graphs, while the corresponding results obtained using the Bandpass filter are illustrated with red bar graphs. (**b**) It showcases the comparison of absolute error in significant wave period. The *x*-axis still represents the experiment groups, and the *y*-axis represents the absolute error in significant wave period (unit: s). Blue bar graphs represent the absolute error in significant wave period calculated using the Sage–Husa adaptive Kalman combined filter, and red bar graphs represent the corresponding results obtained using the Bandpass filter.

As depicted in Figure 12, we have conducted a detailed comparison of the relative error in significant wave height and the absolute error in significant wave period, computed using the Bandpass filter and the Sage–Husa adaptive Kalman combined filter.

The average relative error of effective wave height was 4.40%, with the maximum relative error being 9.64%. The average absolute error of the effective wave period was 0.23 s, with the maximum absolute error being 0.50 s.

According to the “People’s Republic of China National Standard—Seashore Observation Specification”, the error of the effective wave height for Level Ⅰ accuracy should be less than ±10%, and the error of the effective wave period should be less than ±0.5 s. After experimental verification, the wave gauge equipped with the Sage–Husa adaptive Kalman composite filter algorithm met the Level Ⅰ accuracy requirements stipulated in the verification procedure.

For experiments with the same parameters, the wave gauge’s measurement results have good reproducibility, indicating that the filter analysis system has certain stability for wave observation.

For the same original turntable experiment data, when applying the Bandpass filter with fixed upper and lower cutoff frequencies (specifically set to 0.05–0.5 Hz in these experiments), the calculated average relative error in significant wave height was 8.58%, the maximum relative error was 14.76%, the average absolute error in significant wave period was 0.30 s, and the maximum absolute error was 0.50 s.

According to the “National Standard of the People’s Republic of China—Coastal Observation Specifications”, the level II accuracy of significant wave height error should be less than ±15%, and the error in significant wave period should be less than ±0.5 s. Through experimental verification, the wave meter equipped with the Bandpass filtering algorithm meets the level II accuracy stipulated in the verification procedure.

**Figure 13:**
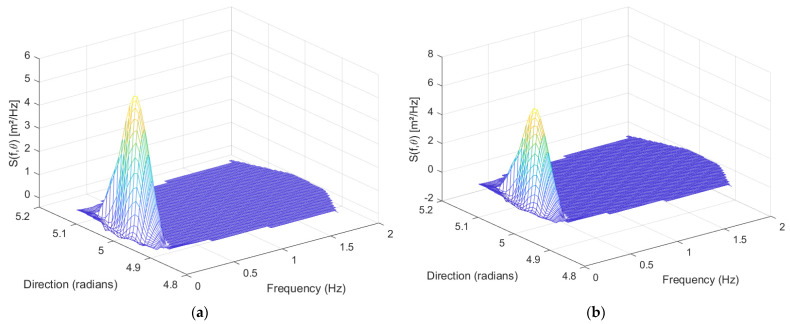
(**a**) It displays a sea wave direction spectrum obtained through the Sage–Husa adaptive Kalman combination filter, depicting the energy distribution of sea waves at different frequencies and directions. In this figure, the *x*-axis represents the direction angle of the sea wave (unit: radian), the *y*-axis represents the sea wave frequency (unit: Hz), and the *z*−axis represents the energy density (unit: m^2^/Hz). (**b**) It presents the sea wave direction spectrum obtained from the same set of data after Bandpass filtering.

As shown in Figure 13, this is a set of wave direction data calculated from the original *x*-axis and *y*-axis acceleration data and angular velocity data obtained from the turntable experiments. This set of direction data, along with the wave displacement data calculated from the *z*-axis acceleration data through the Sage–Husa adaptive Kalman filter, yield the corresponding wave direction spectrum.

Comparing Figure 13a,b, it can be observed that the Sage–Husa adaptive Kalman combination filter retains more sea wave energy. However, once the upper and lower frequency limits of the Bandpass filter are determined, they are not easy to modify in real-time. Hence, it is very likely that some vital energy information will be lost in actual sea conditions.

## 6. Conclusions and Discussion

Through conducting wave simulation experiments with a wave simulation device, we computed errors by comparing the amplitude and period values of standard sinusoidal functions. Through the analysis of experimental data with identical parameters, it was determined that the Sage–Husa adaptive Kalman filter algorithm significantly outperforms the bandpass filter algorithm. More precisely, compared with the average relative error of 8.58% in the effective wave height obtained by the bandpass filter algorithm, the Sage–Husa adaptive Kalman filter reduced it to 4.40%, thereby enhancing the precision of the effective wave height by 48.72%. In addition, relative to the average absolute error of 0.30 s in the effective wave period determined by the bandpass filter algorithm, the Sage–Husa adaptive Kalman filter algorithm reduced it to 0.23 s, consequently improving the accuracy of the effective wave period by 23.33%.

The wave gauge used in the experiment is an accelerometer-based wave gauge developed in-house, built upon MEMS attitude sensors. Its stability and reliability have been validated through sea trials. Future research can delve deeper in the following two aspects:

- Optimizing the circuit of the wave buoy to enhance its anti-interference capability and measurement accuracy in terms of hardware;
- Optimizing the wave direction measurement algorithm. Traditional wave direction measurement algorithms do not correct for abnormal values in the buoy tilt angle data after data readout, and after wave period division, all waves are chosen for wave direction calculation. Thus, the computed main wave direction values are not precise enough, which ultimately impacts the solution of the wave direction spectrum.

In summary, this research provides new perspectives and methods for improving the measurement accuracy of wave gauges and the calculation precision of wave direction spectra, offering significant guidance for future research in related fields.

## Figures and Tables

**Table 1:** Numerical Analysis of Simulation Experiments.

Method	Wave Height (m)	Wave Period (s)
Origin Displacement	4.83	3.90
Bandpass Filter	4.28	4.30
Kalman Filter	4.76	3.88

**Table 2:** Turntable experiment design.

Index	Wave Height (m)	Wave Period (s)
Group 1	1.10	5.65
Group 2	1.10	7.60
Group 3	1.10	9.36
Group 4	2.10	6.25
Group 5	2.10	8.89
Group 6	2.10	10.40
Group 7	3.00	6.13
Group 8	3.00	8.54
Group 9	3.00	10.13
Group 10	4.00	6.24
Group 11	4.00	8.80
Group 12	4.00	10.45

## Data Availability

All the data used in this study were obtained from laboratory measurements. Please contact the author’s email if there is a need to share the experimental data.
